# Connectionist technique estimates of hydrogen storage capacity on metal hydrides using hybrid GAPSO-LSSVM approach

**DOI:** 10.1038/s41598-024-52086-4

**Published:** 2024-01-17

**Authors:** Sina Maghsoudy, Pouya Zakerabbasi, Alireza Baghban, Amin Esmaeili, Sajjad Habibzadeh

**Affiliations:** 1 Surface Reaction and Advanced Energy Materials Laboratory, Department of Chemical Engineering, Amirkabir University of Technology (Tehran Polytechnic), PO Box 15875-4413, Tehran, Iran; 2 Chemical Engineering Department, Amirkabir University of Technology (Tehran Polytechnic), Mahshahr Campus, Mahshahr, Iran; 3 Department of Chemical Engineering, School of Engineering Technology and Industrial Trades, College of the North Atlantic - Qatar, Doha, Qatar

**Keywords:** Energy science and technology, Engineering, Materials science, Mathematics and computing

## Abstract

The AB_2_ metal hydrides are one of the preferred choices for hydrogen storage. Meanwhile, the estimation of hydrogen storage capacity will accelerate their development procedure. Machine learning algorithms can predict the correlation between the metal hydride chemical composition and its hydrogen storage capacity. With this purpose, a total number of 244 pairs of AB_2_ alloys including the elements and their respective hydrogen storage capacity were collected from the literature. In the present study, three machine learning algorithms including GA-LSSVM, PSO-LSSVM, and HGAPSO-LSSVM were employed. These models were able to appropriately predict the hydrogen storage capacity in the AB_2_ metal hydrides. So the HGAPSO-LSSVM model had the highest accuracy. In this model, the statistical factors of R^2^, STD, MSE, RMSE, and MRE were 0.980, 0.043, 0.0020, 0.045, and 0.972%, respectively. The sensitivity analysis of the input variables also illustrated that the Sn, Co, and Ni elements had the highest effect on the amount of hydrogen storage capacity in AB_2_ metal hydrides.

## Introduction

With fossil fuels making up more than 80% of today's energy sources, they are a double-edged sword. On the one hand, a thriving economy has resulted from the heavy usage of fossil fuels, particularly petroleum. On the other hand, due to their limited deposits, fossil fuels are a typical non-renewable resource that will eventually run out^1,2^. As a matter of fact, fossil fuel consumption has left us with ecological problems such as global warming, climate change, and Ozone layer destruction^3–5^. Hence, humankind has turned to clean energy.

According to the European Commission, hydrogen is an energy carrier having great potential for clean, efficient power in stationary, portable, and transport applications^6^. Hydrogen has an energy density three times larger than fossil fuels^7–9^. Various production sources are another reason why hydrogen is an excellent alternative to fossil fuels^10–12^. Hydrogen can be applied to produce energy in the forms of heat or electricity in combustion engines, fuel cells, or turbines, leaving water vapor as a by-product^1,13–15^. Although hydrogen has many advantages, hydrogen storage remains a challenge in the hydrogen economy^1,16–18^. As hydrogen has a low energy density by volume (about 10.7 kJ L^-1^ at ambient conditions), it may need large containers to store hydrogen^10,13^. Academic and industrial communities aim to make hydrogen applicable for practical use by raising the hydrogen storage density as high as possible^8,16^.

Several techniques have been proposed for hydrogen storage^13,14^. Solid-state hydrogen storage technologies, especially metal hydrides, have advantages over other methods due to lower hydrogen storage pressure and release rate^14^. Furthermore, metal hydrides can densely store hydrogen in moderate temperature and pressure conditions^1,13,19^. Metal hydride materials have some essential features such as fast hydrogen absorption and desorption kinetics, the ability to tolerate poisonous materials and contaminations in the hydrogen feedstock, produced efficiently with minimum cost, easy activation, and stability^16,20–23^.

Metal Hydrides are classified into elemental, intermetallic, and complex hydrides^14^. The commonly used hydrogen-storing hydrides are classified based on their stoichiometries; They include but are not limited to AB_5_-type, AB_2_-type, A_2_B-type, and AB-type^7,14,20^. In these structures, A and B are metals with high and low affinity for hydrogen, respectively. The former can typically be a rare-earth or alkaline earth metal (Ca, Ti, Zr, Ta, etc.), while the latter can be a transition metal (Cr, Mn, Co, Fe, Ni, etc.)^7,14,24^. A stable metal hydride formation by element A releases significant energy and increases the temperature during the adsorption process. Alloying A with an element B, which forms an unstable hydride, results in adjustable temperature and pressure conditions, which are desirable traits^8^. AB_2_ metal hydrides are the best choice for room-temperature hydrogen storage as they have fast kinetics, easy activation, and favorable pressure conditions^5,25^. The element selection for the A-site and B-site of AB_2_ compounds determines their hydrogen storage capacity. Thus several studies were carried out on AB_2_ metal hydrides' hydrogen uptake, modifications, and their effects on hydrogen storage density^26–28^. Selecting the A and B elements by experimental methods requires lots of money, energy, and time. Since the required datasets already exist, algorithmic modeling techniques, including machine learning, may become handy in many cases^24^.

Algorithmic modeling culture is now widely used in statistics and data analysis^29–32^. Unlike the traditional approach that uses a stochastical model, the algorithmic culture uses a complex algorithm^33,34^. It gives the new approach the power to significantly speed up calculations, identify intricate systems to decrease prediction errors and make the best possible decisions based on complete status data^35,36^. Moreover, a model derivation is complicated and sometimes impossible, especially when dealing with complex, non-linear systems^7,33,37,38^.

Machine learning has played an undeniable role in advancing research in the fields of energy materials and clean energy. For instance, the relationship between LiBH_4_-mixtures' hydrogen release amount and other factors, such as mixing conditions and operational variables, was predicted by Ding et al.^39^ using gradient boosting regression trees, random forest, Ada decision tree, and decision tree. It was found that temperature was the most important factor^39^. In another study, the effect of pressure, BET surface area, oxygen content, pore volume, and pore size distribution on carbon materials hydrogen uptake was predicted using random forest by Kusdhany et al.^40^. Hydrogen storage in MOFs was predicted by Ahmed and Siegel^41^ using 14 machine learning algorithms, including decision tree, boosted decision tree, support vector machine, etc. 8282 MOFs were claimed to have the potential for reasonable hydrogen storage and were recommended as targets for synthesis^41^.

In recent years, an increasing tendency toward studying machine learning applications in metal hydrides has happened. A database on hydrides for hydrogen storage was examined using linear regression, neural network, Bayesian linear regression, and boosted decision tree by Rahnama et al.^42^. Variables were ranked according to their importance for estimating hydrogen storage capacity. The results showed that boosted decision tree regression outperformed other algorithms, earning a coefficient of determination of 0.83^42^. In another study, multiclass logistic regression, multiclass decision forest, multiclass decision jungle, and multiclass neural network were applied to forecast the ideal metal hydride material class based on the hydrogen weight percent, the heat of formation, and operating temperature and pressure. The deployed models' respective accuracy values were 0.47, 0.60, 0.62, and 0.80^43^. The effects of the reaction chamber's material and shape on the hydrogen adsorption and desorption rates were examined by Wang and Brinkerhoff^21^. The adsorption and desorption efficiency of cylindrical LaNi_5_ hydride beds were predicted using empirical correlations as well as the Radial Basis Neural Network (RBNN). The absolute maximum errors for empirical correlation for adsorption efficiency and desorption efficiency were 8.0% and 6.6%, respectively. The RBNN offered maximum deviations with values below 1.9% and 2.5% for adsorption and desorption efficiencies, respectively^21^. In another study by Suwarno et al.^8^, the heat of formation (ΔH), phase abundance, hydrogen capacity of the AB_2_ alloys, and the impact of the alloying constituents on hydrogen storage properties were examined. The random forest model accurately predicted each hydrogen storage characteristic with an average R^2^ value of 0.722^8^. The hydrogen absorption energy was estimated using a robust Gaussian process regression (GPR) method with four kernel functions by Gheytanzadeh et al.^7^. All of the GPR models performed exceptionally well; however, the GPR with an exponential kernel function had the highest precision, as measured by R^2^, MRE, MSE, RMSE, and STD, which were, respectively, 0.969, 2.291%, 3.909, 2.51, and 1.878. The analysis's sensitivity revealed that Zr, Ti, and Cr are the system's most demining components^7^. The aforementioned study paved the way for further investigation of hydrogen storage capacity in AB_2_ metal hydrides.

While several machine learning studies have explored various facets of metal hydrides, including adsorption/desorption thermodynamics, the current study stands out by employing innovative hybrid ML techniques—a rarity in machine learning studies within the realm of chemical engineering. This distinctive approach enhances the comprehensiveness and novelty of our investigation beyond existing studies in the field. In this study, three machine learning algorithms including GA-LSSVM, PSO-LSSVM, and HGAPSO-LSSVM are employed with the aim of the prediction of hydrogen storage capacity in the AB_2_ metal hydrides. Utilizing the database obtained from the literature, 22 alloying elements are considered as the model's input variables. In such a manner, the correlation between the chemical components and hydrogen storage capacity in AB_2_ metal hydrides is calculated. Statistical factors including R^2^, STD, MSE, RMSE, and MRE are determined to evaluate the accuracy of the developed models. Also, by performing a sensitivity analysis for each input variable, the most effective parameters on the hydrogen storage capacity are identified.

## Methodology

### Predictive models

The least square support vector machine (LSSVM) method is utilized in the present study as an extended support vector machine (SVM) method. To improve the computation speed and accuracy, the LSSVM method transfers the two-dimensional programming of the SVM method into a linear space^44–46^. The penalty factor and kernel parameters are optimized using the genetic algorithm (GA), the particle swarm optimization (PSO), and the hybrid of GA and PSO (HGAPSO) to further improve the classification accuracy. Therefore, three machine learning models including GA-LSSVM, PSO-LSSVM, and HGAPSO-LSSVM are developed in the present study, and their ability to estimate the amount of hydrogen storage capacity in various AB_2_ metal hydrides is examined.

#### Least squares support vector machine (LSSVM)

The LSSVM method is relatively a new supervised learning model. Based on the first type of SVM, Suykens and Vandewalle^47^ presented this method in 1999. With this method, learning algorithms are employed to examine data and identify its pattern. Classification, pattern recognition, and regression problems can be analyzed using the LSSVM method. This method is established based on statistical learning theories (SLT)^48^. Compared with the traditional SVM method, the LSSVM method has higher generalization capability, lower computational complexity, and higher running speed^49^. The general equation of the LSSVM method can be presented based on Eq. (1) ^50^.

1 $$f(x) = \omega^{T} \phi + b$$

where *f* depicts the connection between the target and input variables, *ω* is the weight vector, *ϕ* is the mapping function, and *b* is the bias term.

The amounts of weight vector and bias term are estimated based on the objective function presented in Eq. (2). Also, the associated constraint is illustrated in Eq. (3) ^50^.

2 $$\min_{\omega ,\,b\,,\,e} J(\omega ,\,e) = \frac{1}{2}||\omega ||^{2} + \frac{1}{2}\gamma \sum\nolimits_{i = 1}^{n} {e_{i}^{2} }$$

3 $$y_{i} = \omega^{T} \,\phi (x_{i} ) + b + e_{i} \,\,\,\,\,\,\,\,\,\,{\text{i = 1,}}\,{2,}\,.....{,}\,{\text{m}}$$

where *ω* is the weight vector, *b* is the bias term, *ϕ* is the mapping function, *e*_*i*_ is the training error of *x*_*i*_, and *γ* is the regularization factor.

The final regression form of the LSSVM method for function estimation can be expressed according to Eq. (4) ^51–54^.

4 $$f(k) = \sum\limits_{k = 1}^{N} {\alpha_{k} K(x,\,x_{k} )} + b$$

where *a*_*k*_ is the Lagrange multiplier, *b* is the bias term, and *K (x, x*_*k*_*)* is the radial basis function (RBF) kernel which is presented in Eq. (5) ^51,52,54^.

5 $$K(x,\,x_{k} ) = \exp \left( { - \frac{{||x_{k} - x||^{2} }}{{\sigma^{2} }}} \right)$$

where *σ*^*2*^ represents the squared bandwidth that can be optimized utilizing GA, PSO, or HGAPSO algorithms during the calculation process.

Equation (6) demonstrates the mean square error (MSE) as the objective function of the optimization method^50,55^.

6 $$MSE = \frac{{\sum\limits_{i = 1}^{N} {(HSC_{pred} - HSC_{\exp } )^{2} } }}{N}$$

where *N* is the number of datapoints and *HSC*_*pred*_ and *HSC*_*exp*_ represent the predicted and experimental hydrogen storage capacity, respectively.

The optimization problem can be defined as Eq. (7) ^50,55^.

7 $$\min \,F(\gamma ,\,\sigma^{2} ) = \min \,(MSE)$$

where *γ* is the regularization factor, *σ*^*2*^ represents the squared bandwidth, and *MSE* denotes the mean square error.

**Figure 1:**
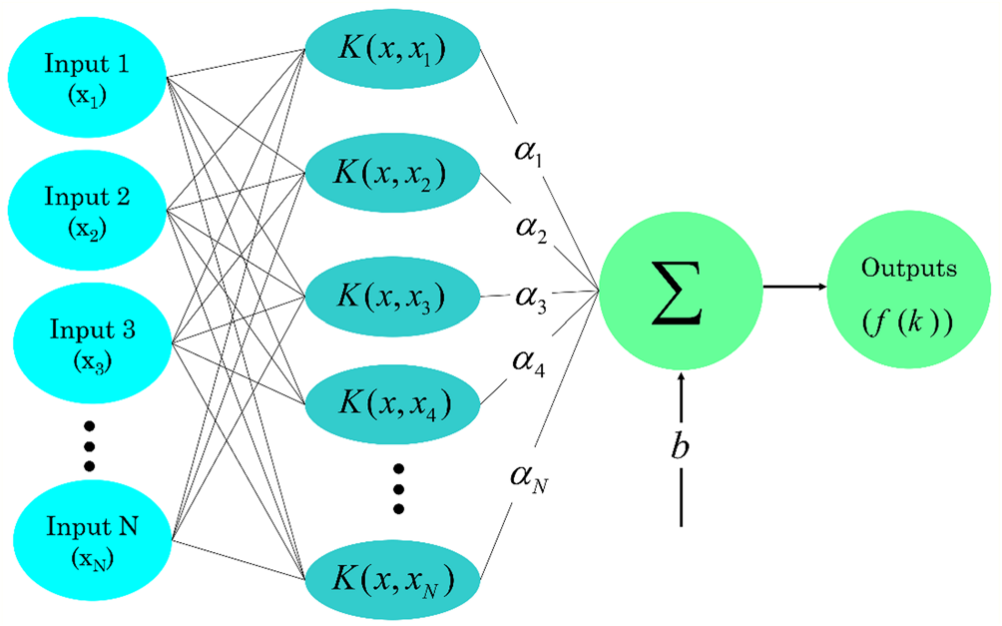
The associated structure of the LSSVM model.

Figure 1 demonstrates the associated structure of the LSSVM model.

#### Genetic algorithm (GA)

**Figure 2:**
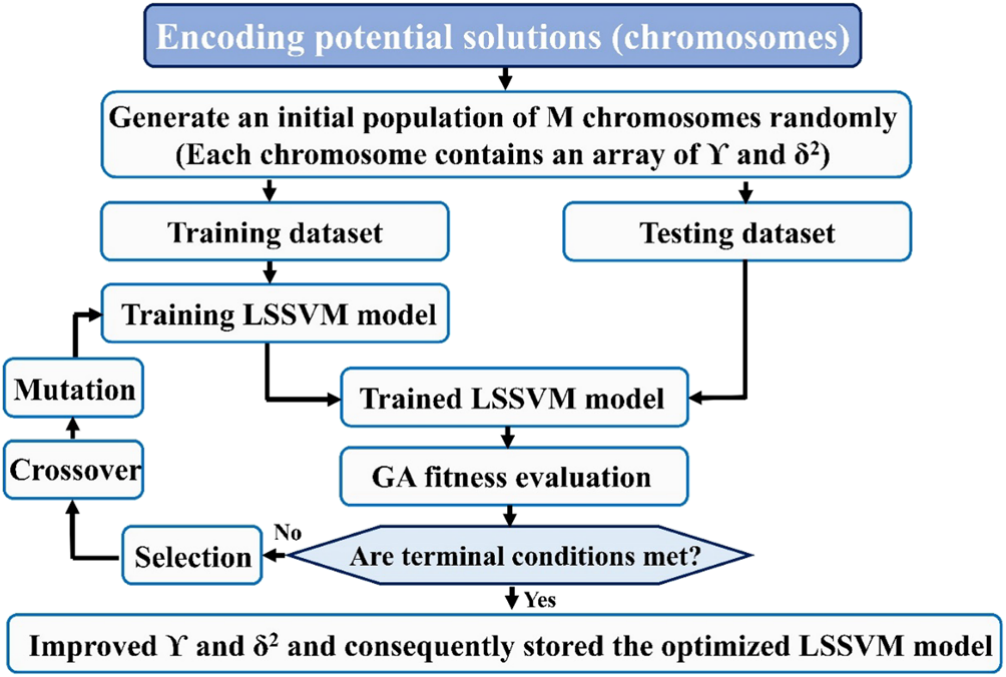
The schematic diagram of the GA-LSSVM model.

In the 1970s, Holland^56^ first proposed the genetic algorithm as a computational model. This algorithm that originates from genetic and natural selection is one of the popular optimization methods to simulate the genetic mechanism of Darwinian biological evolution and natural selection^49^. By simulating the natural evolution process, the genetic algorithm is a technique for determining the optimal solution. Stochastic transition criteria and exploration in the solution space are used in the genetic algorithm method. The generation of the initial population, choosing the GA operator, and the evaluation are three significant steps in this method^44^. Chromosomes, the algorithm's initial solution, are randomly created using a variety of operators including crossover, mutation, and reproduction. In the form of genes with *γ* and *σ*^*2*^ as two parameters, each chromosome contains the solution. Also, for the definition of offspring production probability, the crossover factor (CF) and mutation factor (MF), which represent the probability of changing chromosomes situation, can be utilized^50^. The schematic diagram of the GA-LSSVM model is illustrated in Fig. 2.

#### Particle swarm optimization (PSO)

Kennedy and Eberhart^57^ initially presented the PSO algorithm as an evolutionary computing method. Nowadays, the PSO method is frequently utilized in machine learning applications including neural networks, fuzzy control, and functional optimization^58–61^. PSO algorithm has fewer adjustment parameters than other optimization techniques as well as the convergence speed is fast, and it is straightforward and simple to implement^54^. The PSO algorithm is developed by the behavior of social organisms like a flock of birds. The procedure starts with the generation of random solutions, called particles. The generations are then modified to find the optimal solution. The set of particles, called swarm, moves throughout the search space with a flexible velocity and retains the best location it has found. Each particle is capable of modifying its velocity vector to find the optimal location^44,51,54^. Particle updating velocity can be formulated according to Eq. (8) ^48,50,53^.

8 $$v_{id} (t + 1) = wv_{id} (t) + c_{1} r_{1} (p_{best,\,id} (t) - X_{iid} (t)) + c_{2} r_{2} (g_{best,\,d} (t) - X_{id} (t))\,\,\,\,\,\,\,\,\,\,{\text{d = 1,}}\,{2,}\,...{,}\,{\text{D}}$$

where *v*_*i*_ is the velocity vector, *t* is the time instant, *X*_*i*_ is the position vector, *p*_*best, id*_ is the best previous position of particle i, *g*_*best, id*_ is the best global position of particle i, *w* is the inertia weight, *c* is the learning rate, and *r* is the random number.

**Figure 3:**
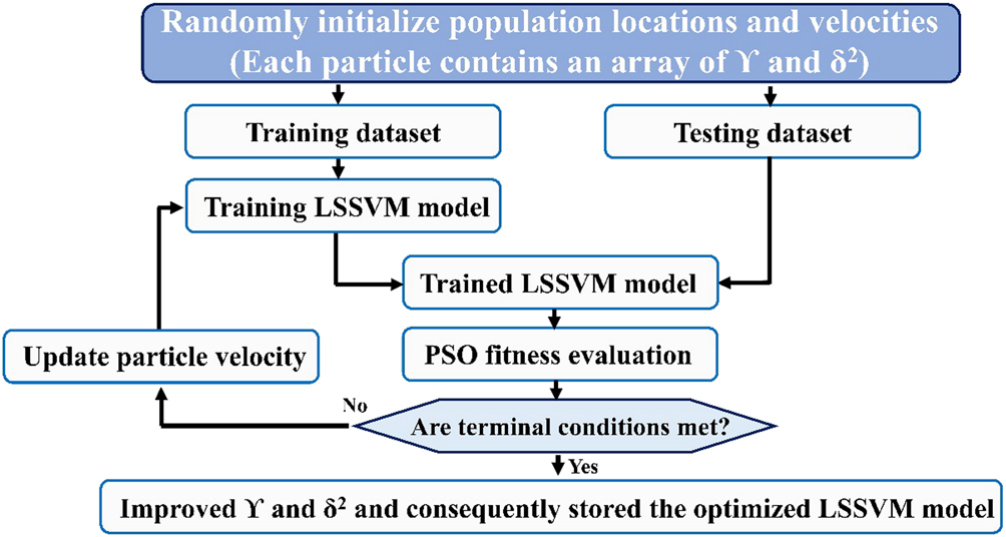
The schematic diagram of the PSO-LSSVM model.

The new particle location is equal to the sum of the new velocity and the prior particle location. It can be formulated based on Eq. (9) ^48,50,53^.

9 $$X_{id} (t + 1) = X_{id} (t) + v_{id} (t + 1)\,\,\,\,\,\,\,\,\,\,\,{\text{d = 1,}}\,{2,}\,...{,}\,{\text{D}}$$

where *X*_*i*_ is the position vector, *t* is the time instant, and *v*_*i*_ is the velocity vector. The schematic diagram of the PSO-LSSVM model is illustrated in Fig. 3.

#### Hybrid GA and PSO (HGAPSO)

**Figure 4:**
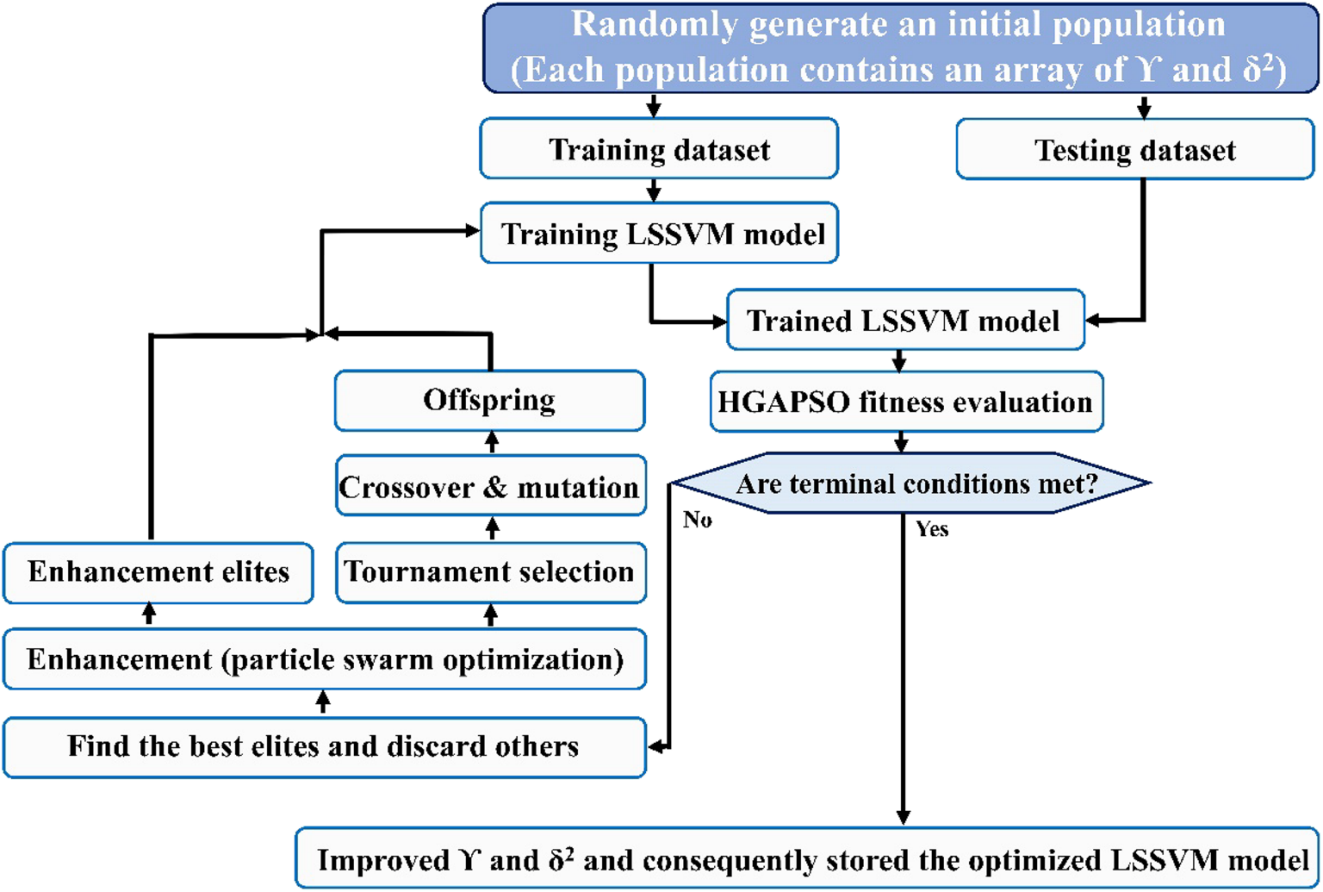
The schematic diagram of the HGAPSO-LSSVM model.

Juang^62^ proposed the idea of combining the genetic algorithm with the particle swarm optimization. Although the GA can be utilized for a variety of problems, large-scale optimization issues including time and cost-consuming computation are observed in this algorithm. So, to utilize the benefits and abilities of both optimization algorithms, GA and PSO are combined. A population with the new features of offspring and improved elites is obtained by the combination of the GA and PSO algorithms^44,50,52^. The schematic diagram of the HGAPSO-LSSVM model is illustrated in Fig. 4.

### Data collection

In the present study, the total number of 244 AB_2_ alloy datasets were collected from the literature^8^. The collected data is presented in the supplementary information. The AB_2_ alloying elements, and hydrogen storage capacity (wt.%) are provided in the dataset. To estimate the hydrogen storage capacity in the AB_2_ metal alloys, 22 alloying elements including Mg, Zr, Ce, Mn, Sn, Mo, Cr, V, Ni, Fe, La, Al, C, Ti, Gd, B, Si, W, Cu, Co, Nb, and Ho are considered as the input variables. Meanwhile, the associated hydrogen storage capacity is utilized as an output variable. To attain a highly accurate model, 25% of the total data was separated randomly as the testing dataset while the remaining 75% of the data was employed for training the model.

### Model evaluations

R^2^, standard deviation (STD), mean-square error (MSE), root-mean-square error (RMSE), and mean relative error (MRE) are the statistical factors that can be used to quantify the developed model accuracy^63–65^. The respective factors are illustrated in Eqs. (10)–(14).

10 $$R^{2} = 1 - \frac{{\sum\limits_{i = 1}^{n} {[x_{i}^{predicted} - x_{i}^{\exp erimental} ]^{2} } }}{{\sum\limits_{i = 1}^{n} {[x_{i}^{predicted} - x_{m} ]^{2} } }}$$

11 $$STD = \sqrt {\sum\limits_{i = 1}^{n} {\frac{{[x_{i}^{predicted} - x_{m} ]^{2} }}{n}} }$$

12 $$MSE = \frac{1}{n}\sum\limits_{i = 1}^{n} {[x_{i}^{predicted} - x_{i}^{\exp erimental} ]^{2} }$$

13 $$RMSE = \sqrt {\frac{{\sum\limits_{i = 1}^{n} {[x_{i}^{predicted} - x_{i}^{\exp erimental} ]^{2} } }}{n}}$$

14 $$MRE = \frac{1}{n}\frac{{|x_{i}^{predicted} - x_{i}^{\exp erimental} |}}{{x_{i}^{\exp erimental} }}$$

where *n* is the number of datapoints and *m* denotes the mean value.

### Model development

As already described, 22 input variables including alloying elements were utilized to estimate the hydrogen storage capacity of AB_2_ metal hydride alloys as the target parameter. In the present study, the developed GA-LSSVM, PSO-LSSVM, and HGAPSO-LSSVM models presented above are employed using the package MATLAB R2022b which is widely used in soft modeling and machine learning problems. The hardware with the 5 cores CPU model and 12 GB of RAM was utilized in all simulations. The respective computational times were about 118, 136, and 167 s for the GA-LSSVM, PSO-LSSVM, and HGAPSO-LSSVM models. It should be mentioned that the difference in computational times is not sufficiently notable to be comparable.

## Results and discussion

### Outlier analysis

It is always conceivable that the dataset contains some outlier data. Due to various reasons including the degree of accuracy, research assumptions, and instrumental or human errors, these data might be included in the dataset. The behavior of the outlier data differs from that of the other datapoints, which causes errors in the machine learning algorithms. Therefore, these data must be eliminated as suspected data from the training and testing procedure^66^. Various methods have been proposed so far to separate the outlier data. One of the most well-known and widely used of these methods is the leverage method. Based on this method, the Hat matrix is obtained according to Eq. (15) ^66^.

15 $$H = U(U^{t} U)^{ - 1} U^{t}$$

where *H* is the Hat matrix, *U* is the (*n* × *p)* matrix, *n* is the number of datapoint, *p* is the number of model parameters, and *t* is the transpose matrix.

As illustrated in Eq. (15), the Hat value (*HV*) for each data is equal to the corresponding diagonal element in the Hat matrix. Based on this method, the critical leverage limit is determined according to Eq. (16) ^66^.

16 $$H^{*} = \frac{3 \times (p + 1)}{n}$$

where H^*^ is the critical leverage limit, *n* is the number of datapoint, and *p* is the number of model parameters.

**Figure 5:**
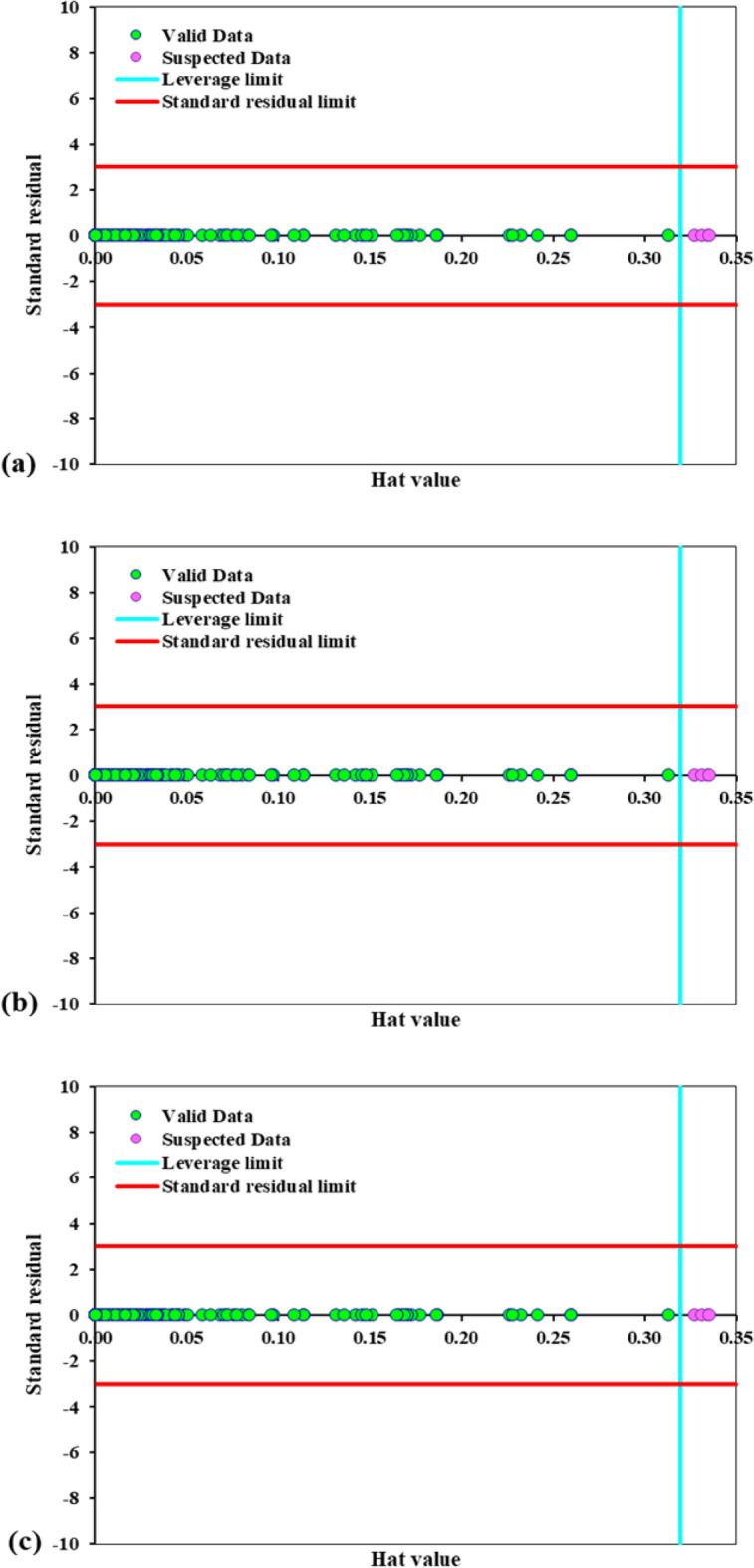
Detection of suspected data based on William’s plots depicted for (**a**) GA-LSSVM, (**b**) PSO-LSSVM, and (**c**) HGAPSO-LSSVM.

Following the calculation of the Hat values and crucial leverage limit based on Eq. (15)–(16), William’s plot is utilized to graphically identify the outlier or suspected data. In this plot, the standardized residual values (*R*), which are defined as the difference between the experimental and the corresponding predicted values, are presented against the Hat values. Based on William’s plot, the data in the range of $0 \le HV \le H^{*}$ and $- 3 \le R \le 3$ have acceptable accuracy. The data located outside of these ranges are identified as the outlier or suspected data and are excluded from the training and testing procedures. It can be clearly seen from Fig. 5 that most of the data relevant to the hydrogen storage capacity are located within the valid range. The identified outlier data for all models is 15. This demonstrates that the dataset obtained from the literature can be well utilized for training and testing the three machine learning models considered in the present study.

### Sensitivity analysis

Sensitivity analysis is employed to quantify the effect of each input parameter on the variation of the output parameter^67,68^. In this analysis, a relevancy factor (*r*) is calculated for each input parameter based on Eq. (17).

17 $$r = \frac{{\sum\limits_{i = 1}^{n} {(X_{k,\,i} - \overline{X}_{k} )(Y_{i} - \overline{Y} )} }}{{\sqrt {\sum\limits_{i = 1}^{n} {(X_{k,\,i} - \overline{X}_{k} )^{2} } \sum\limits_{i = 1}^{n} {(Y_{i} - \overline{Y} )^{2} } } }}$$

where $X_{k,\,i}$ is the 'i' th data of the 'k' th input variable, $\overline{X}_{k}$ is the data average of the 'k' th input variable, $Y_{i}$ is the 'i' th data of the output variable, $\overline{Y}$ is the data average of the output variable, and *n* is the number of datapoint.

**Figure 6:**
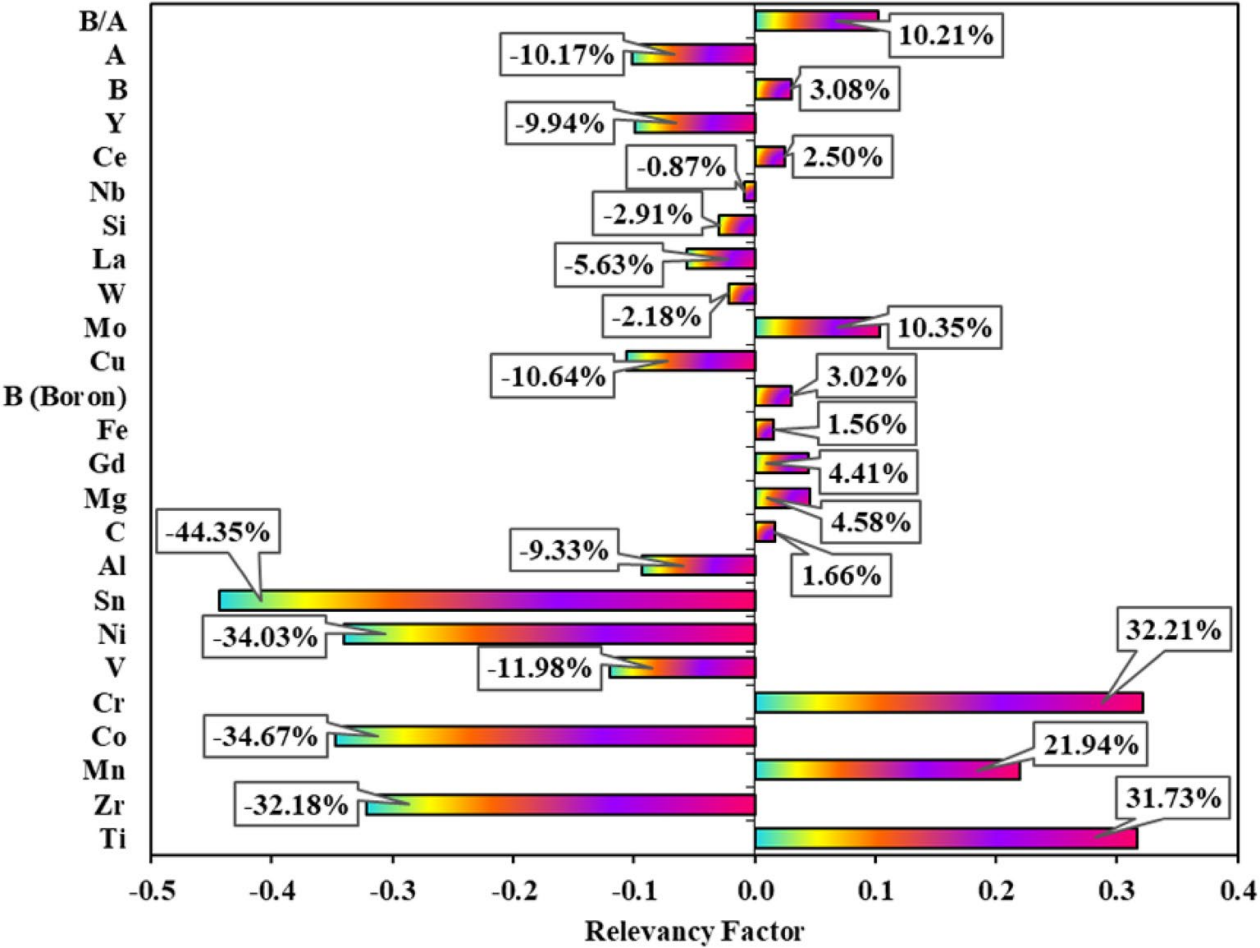
Sensitivity analysis for determining effective variables on the hydrogen storage capacity of AB_2_ metal hydrides.

According to Eq. (17), it can be said that the relevancy factor has a value between −1 and 1. The examined input variable affects the output variable positively when the respective relevancy factor has a positive value. The reverse occurs when this factor has a negative sign. Accordingly, the influence of a parameter on the output variable increases with the increment of the respective relevancy factor regardless of its sign. The effects of the input variables on the amounts of hydrogen storage capacity by calculating the relevancy factor of each parameter are shown in Fig. 6. Based on the obtained results, the amounts of Sn, Co, and Ni elements in the AB_2_ metal hydrides have the greatest impact on the hydrogen storage capacity. The relevancy factors of these elements are 44.65%, 34.67%, and 34.03%, respectively. On the other hand, Nb, Fe, and C with the respective relevancy factors of 0.87%, 0.1.56%, and 0.1.66% have the slightest impact on the hydrogen storage capacity.

### Modeling results and validation

As previously indicated, the LSSVM algorithm contains two tuning parameters. The squared bandwidth (*σ*^*2*^) and regularization factor (*γ*) should be determined by the developed evolutionary algorithms. Based on the calculation, the obtained amounts of these parameters are reported in Table 1.

**Table 1:** Determined hyperparameters of the LSSVM model optimized by the evolutionary algorithms.

Parameters	GA	PSO	HGAPSO
$\sigma^{2}$	1.17594	1.09584	102,426
$\gamma$	9563.8632	9612.5626	9684.7452

The accuracy evaluation of the developed models will be achievable by calculating the statistical factors. These factors are presented in Table 2. Based on the calculation, the amounts of R^2^, STD, MSE, RMSE, and MRE for the training data of the GA-LSSVM model are 0.948, 0.084, 0.0080, 0.089, and 4.305%, respectively. These respective values are 0.953, 0.080, 0.0071, 0.084, and 2.817% for the PSO-LSSVM model and 0.969, 0.066, 0.0048, 0.069, and 2.034% for the HGAPSO-LSSVM model. It can be said that all three employed models are able to fit the training dataset. The capability of these developed models to predict the hydrogen storage capacity in other AB_2_ metal hydrides can be evaluated utilizing the test data. The amounts of statistical factors for the test data of the GA-LSSVM model are 0.927, 0.077, 0.0070, 0.084, and 3.241%, respectively. These respective values are 0.956, 0.060, 0.0043, 0.066, and 2.503% for the PSO-LSSVM model and 0.980, 0.043, 0.0020, 0.045, and 0.972% for the HGAPSO-LSSVM model. According to calculated factors, it can be concluded that all three models proposed in the present study can be used to predict the hydrogen storage capacity in AB_2_ types of metal hydrides. Overall statistical factors for the entire set of training and testing data are also calculated in Table 2. Based on that, it can be claimed that, among the suggested models, the HGAPSO-LSSVM model has the highest accuracy in hydrogen storage capacity estimation.

**Table 2:** Evaluation of the statistical factors of the proposed GA-LSSVM, PSO-LSSVM, and HGAPSO-LSSVM models.

Model	Group	R^2^	MRE (%)	MSE	RMSE	STD
GA	Train data	0.948	4.305	0.007987219	0.0894	0.0839
	Test data	0.927	3.241	0.007005121	0.0837	0.0772
	Total data	0.944	3.114	0.007741694	0.0837	0.0821
PSO	Train data	0.953	2.817	0.007147408	0.0845	0.0798
	Test data	0.956	2.503	0.004319724	0.0657	0.0603
	Total data	0.954	2.726	0.006440487	0.0657	0.0753
HGAPSO	Train data	0.969	2.034	0.00479724	0.0693	0.0662
	Test data	0.980	0.972	0.002025748	0.0450	0.0430
	Total data	0.970	1.727	0.004104367	0.0450	0.0612

**Figure 7:**
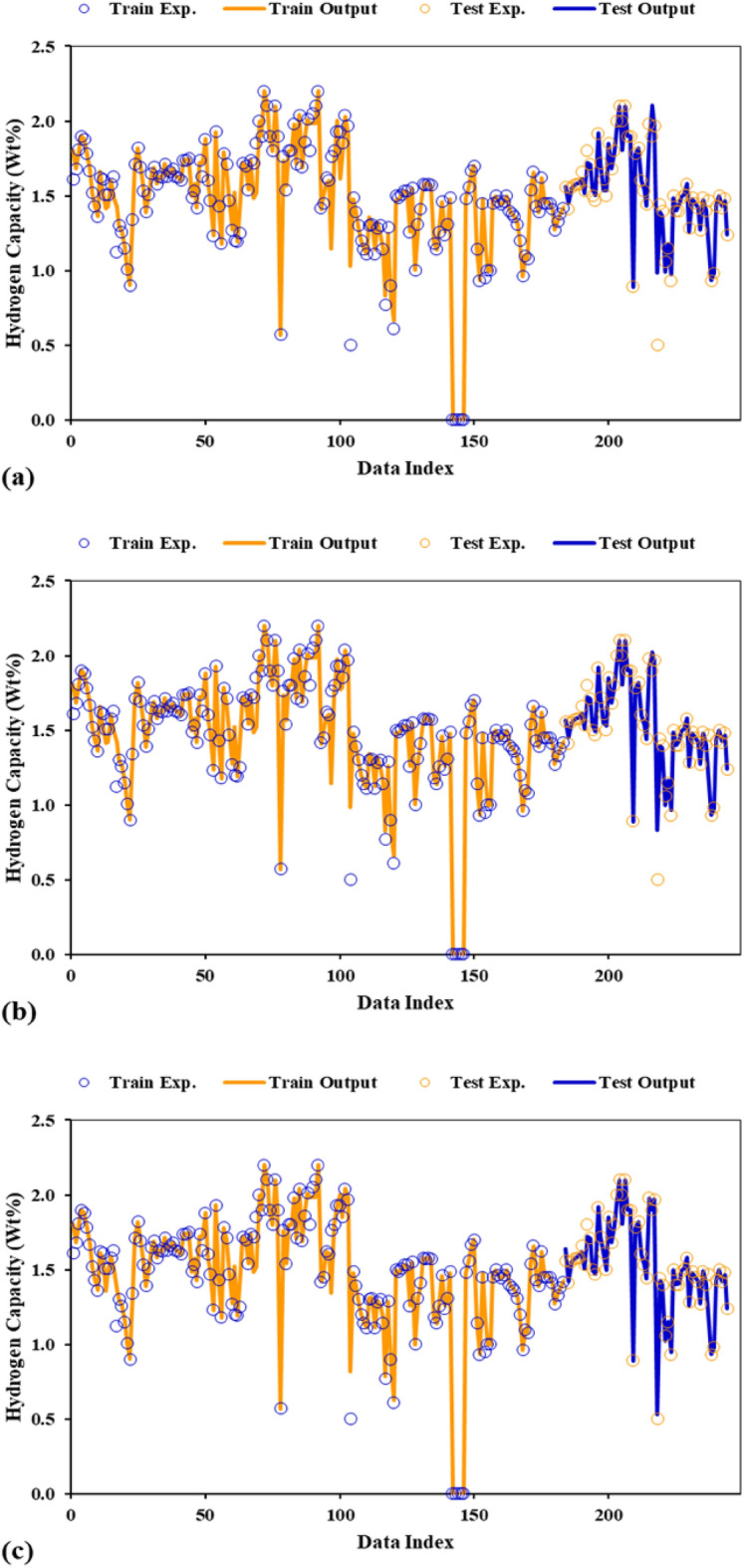
Comparison of actual and predicted values at training and testing stages depicted for (**a**) GA-LSSVM, (**b**) PSO-LSSVM, and (**c**) HGAPSO-LSSVM.

The accuracy of the three proposed models is graphically analyzed in Fig. 7. As can be seen in Fig. 7, the models-based predictive lines match the points which represent the real values. This adaptation is observed in all three developed models and for both training and testing datasets. The results of Fig. 7 provide additional evidence that the proposed models in this study are able to estimate the amount of hydrogen storage capacity in various types of AB_2_ metal hydrides. Utilizing these models will pave the way for obtaining a metal hydride with an optimal hydrogen storage capacity.

**Figure 8:**
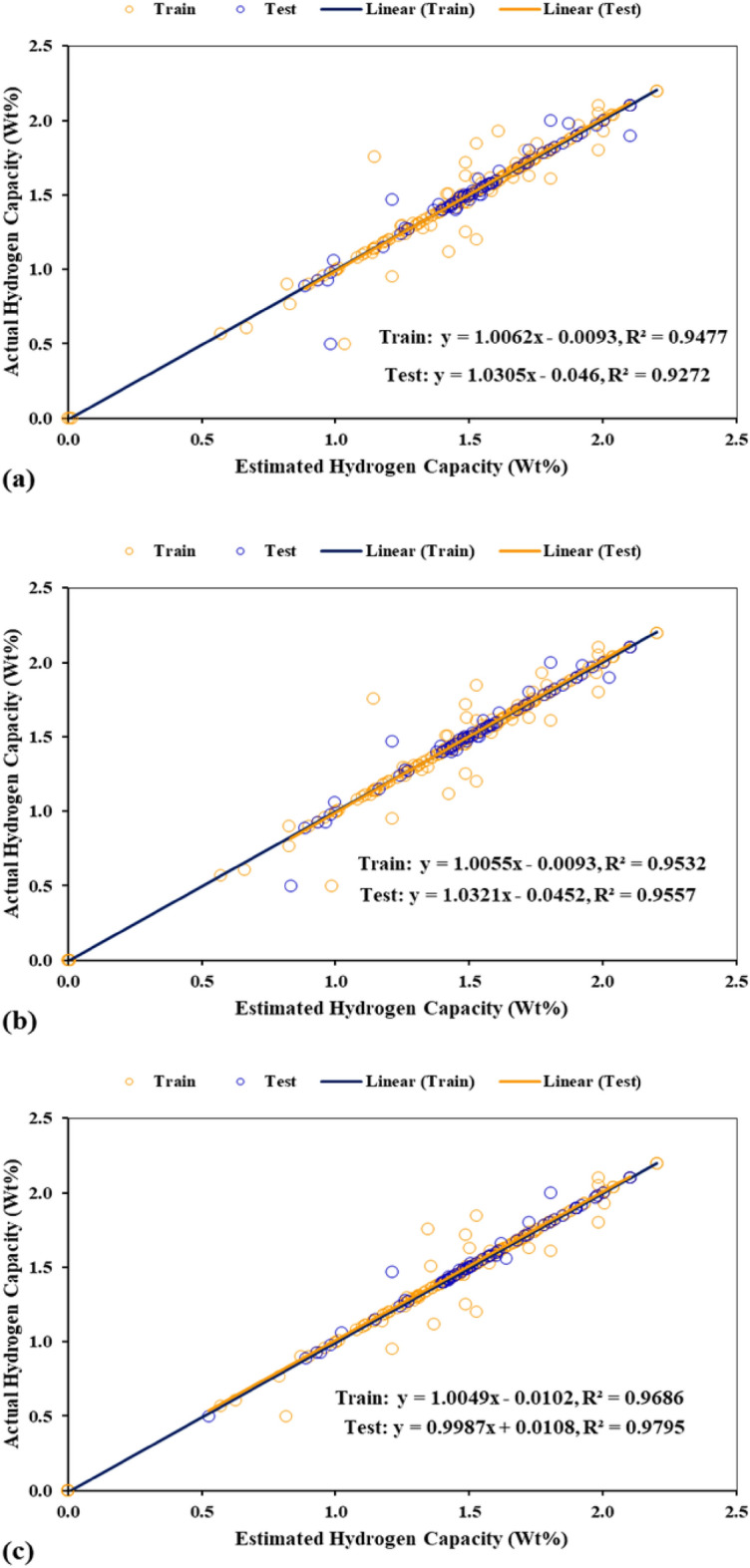
Cross plots of the train and test dataset depicted for (**a**) GA-LSSVM, (**b**) PSO-LSSVM, and (**c**) HGAPSO-LSSVM.

The utilization of cross plots is another method to evaluate the accuracy of developed models. In these plots, the bisector line serves as an indicator for the models' accuracy so that the created model is more accurate whenever the training and test data are closer to this line. The cross plots for the three proposed models are presented in Fig. 8. Based on the obtained results, in all three developed models, the training and test data are located extremely near the bisector line. The linear fitting equations of the training and test data are also presented in Fig. 8. The amounts of R^2^ of these lines for the training data of the GA-LSSVM, PSO-LSSVM, and HGAPSO-LSSVM models are 0.9477, 0.9532, and 0.9686, respectively. These respective values are 0.9272, 0.9557, and 0.9795 for the test data. According to the calculation, it can be inferred that by utilizing the proposed models the hydrogen storage capacity in AB_2_ metal hydride can be properly predicted.

**Figure 9:**
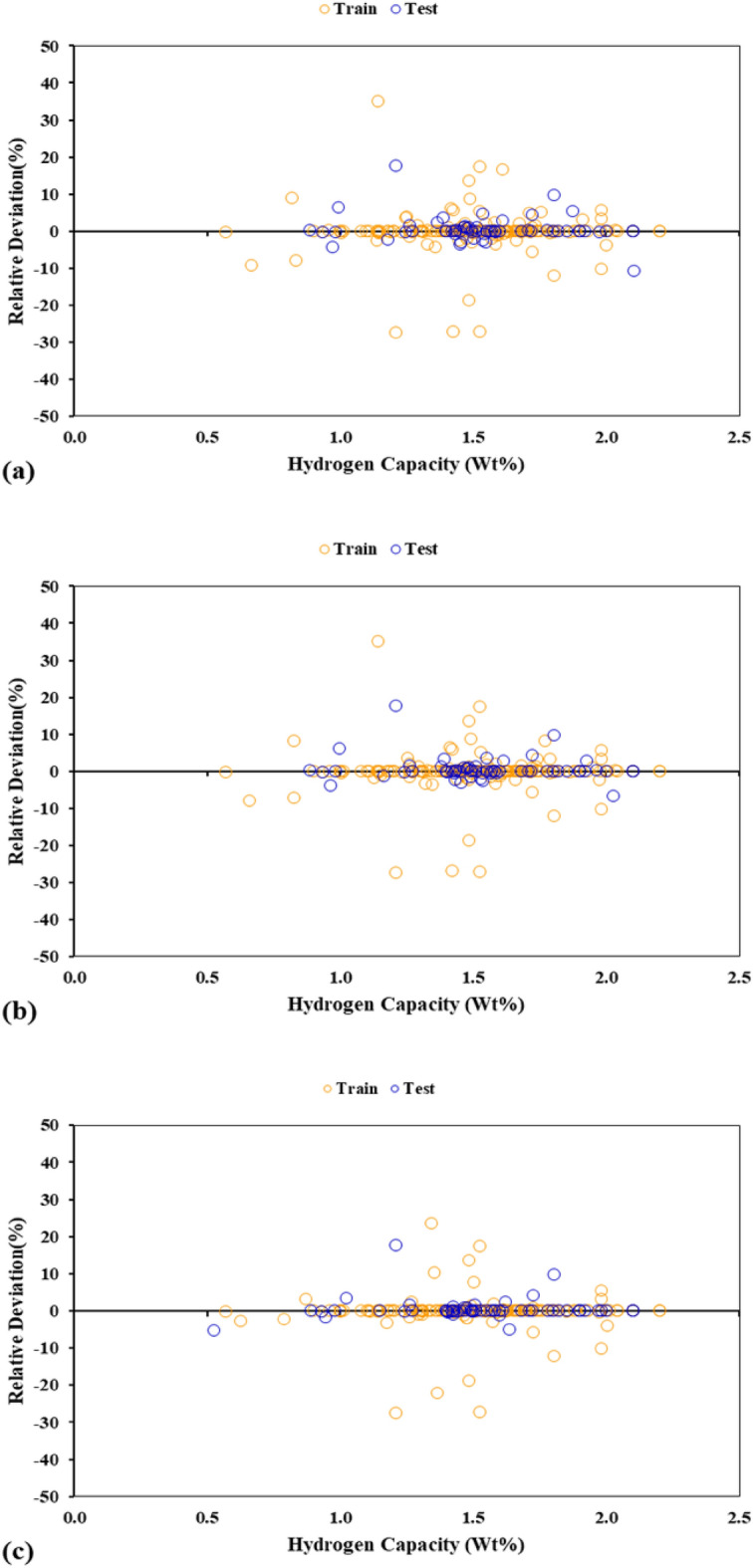
Relative deviation plots of the train and test dataset depicted for (**a**) GA-LSSVM, (**b**) PSO-LSSVM, and (**c**) HGAPSO-LSSVM.

The relative deviation of the actual hydrogen storage capacity and its predicted values are shown in Fig. 9. The majority of the training and test data just have a slight amount of relative deviations which again demonstrates the excellent accuracy of the developed models. The maximum values of relative deviation for the GA-LSSVM, PSO-LSSVM, and HGAPSO-LSSVM models are about 35%, 33%, and 25%, respectively. It can be observed that the HGAPSO-LSSVM has the highest accuracy among the proposed models. Moreover, all three suggested models provide an accurate estimation of the hydrogen storage capacity.

In general, the developed models demonstrated excellent performance for estimating the hydrogen storage capacity in AB_2_ metal hydrides. So, maximizing the hydrogen storage capacity can be facilitated using the proposed models. It should be mentioned that one of the research's issues is the established models are restricted to AB_2_ metal hydrides. Therefore, the foregoing models may encounter shortcomings in the other types of metal hydrate. Accordingly, generating models based on more extensive types of metal hydrate could be another line of future investigation.

## Conclusion

The prediction of hydrogen storage capacity in the AB_2_ metal hydrides was argued in the present study developing three machine learning algorithms including GA-LSSVM, PSO-LSSVM, and HGAPSO-LSSVM. 22 alloying elements were considered as these models' input variables and the correlation between the chemical components and the hydrogen storage capacity was determined. Based on the obtained results, all three utilized models had high accuracy in estimating the amount of hydrogen storage capacity. Among these models, the HGAPSO-LSSVM algorithm had the highest accuracy and was selected as the best model. In this model, the statistical factors of R^2^, STD, MSE, RMSE, and MRE were 0.980, 0.043, 0.0020, 0.045, and 0.972%, respectively. For the PSO-LSSVM model, these respective values were 0.956, 0.060, 0.0043, 0.066, and 2.503%. As well as the respective values of 0.927, 0.077, 0.0070, 0.084, and 3.241% were achieved for the GA-LSSVM model. Based on the performed sensitivity analysis, the amounts of Sn, Co, and Ni elements with the respective relevancy factor of 44.65%, 34.67%, and 34.03% had the highest effect in the variation of hydrogen storage capacity, respectively. The outcomes of the present study can pave the way to achieving the appropriate selection of AB_2_ elements to maximize the hydrogen storage capacity.

### Supplementary Information

Supplementary Information.

## Data Availability

All data generated or analyzed during this study are included in this published article/supplementary information files.
